# Supplementary material for: The Use of Nitrosative Stress Molecules as Potential Diagnostic Biomarkers in Multiple Sclerosis
Source: Int J Mol Sci. 2024 Jan 8;25(2):787. doi: 10.3390/ijms25020787 (PMC10815836; doi:10.3390/ijms25020787)
Supplement: Supplementary file 1 [file ijms-25-00787-s001.zip › Suppl. Table S1.pdf]

Pseudonym	Age	Sex	Duration since 1. symp. (Y)	Duration since 1. diagnosis (Y)	Previous DMTs	Medication	Comorbidities	Relapse	EDSS	MRI active lesions	NOx serum (µM)	NOx CSF (µM)
NIT-7984596	41	f	18.6	18.5	no	no	Vitiligo	no	1.0	n/a	4.1	n/a
NIT-7936476	44	f	3.6	3.5	no	no	n/a	no	0.0	no	0.7	n/a
NIT-7968645	32	f	0.9	0.0	no	no	Migraine with aura, lipedema, obesity	no	1.0	no	3.4	2.7
NIT-7942539	57	f	1.6	0.0	no	Valsartan	S/p TEP of the left knee, s/p spinal surgery for L4/5 prolapse, s/p cholecystectomy, eutyhreotic goiter, s/p removal of a giant cell tumor of the right digitus III	yes	1.0	no	4.9	3.5
NIT-6047403	52	f	0.1	0.0	no	no	Chronic lacunar defect in the posterior limb of the left internal capsule DD Virchow-Robin space	yes	2.0	yes	6.5	n/a
GEN-K-7888842	57	m	0.1	0.6	no	Vitamin D	AHT	no	0.0	yes	9.8	3.3
NIT-7936341	53	m	4.2	0.3	no	Vitamin D	Subclavian stenosis, vitamin D deficiency, right adrenal adenoma, hypercholesterolemia	no	6.0	no	18.4	n/a
NIT-3276531	38	f	0.0	0.0	no	Ramipril, amlodipine, vitamin D	Lipedema, aHT, pineal cyst DD pineocytoma, chronic bronchitis, asthma	yes	2.5	yes	11.9	4.3
NIT-7990398	24	f	0.2	0.2	no	no	no	no	1.0	yes	10.5	n/a
NIT-7968168	54	m	0.7	0.3	no	Vitamin D, sertraline	Cataract, COPD, axonal peripheral neuropathy, aHT, s/p bladder cancer in 2018, anxiety disorder	no	2.5	n/a	35.2	n/a
NIT-7993617	37	f	0.7	0.3	no	no	Neurodermatitis, aHT, hyperlipoproteinemia	no	2.5	no	15.1	n/a
NIT-7985475	39	m	1.7	0.0	no	no	Depressive adjustment disorder	yes	5.0	yes	1.5	n/a
REB/K1-6670439	23	f	0.8	0.6	no	Levetiracetam	Symptomatic epilepsy	no	2.0	n/a	2.2	n/a
NIT-7999590	34	m	0.3	0.2	no	no	Polyneuropathy	no	n/a	yes	1.4	n/a
NIT-7588734	31	f	2.7	2.7	no	no	Hypothyroidism	no	1.0	n/a	3.7	n/a
NIT-4311195	57	f	18.8	0.0	no	no	Disc herniation	no	2.0	yes	4.7	n/a
NIT-7689525	39	f	0.7	0.1	no	no	Depression and anxiety disorder	no	2.0	yes	3.5	n/a
NIT-6567357	57	f	1.8	0.0	no	Fampridine	S/p resection of a bladder carcinoma, hypercholesterolemia	no	2.0	n/a	9.2	n/a
NIT-7909971	49	f	1.1	0.1	no	no	Latent tuberculosis	no	2.0	yes	3.9	n/a
NIT-5950698	36	m	0.6	0.2	no	no	Alcohol use disorder, hip osteoarthritis, h/o epileptic seizure during alcohol withdrawal, steatosis hepatis and pancreatic lipomatosis, coxarthrosis	no	2.5	no	0.9	n/a
NIT-7958373	34	f	1.3	0.3	no	no	H/o disc herniation	no	2.0	no	11.1	3.7
NIT-8058216	28	f	0.5	0.4	no	Folate	no	no	0.0	n/a	1.9	n/a
NIT-8041314	55	f	0.8	0.5	no	no	Asthma, aHT, disc herniation, h/o breast surgery, polyneuropathy	no	2.5	n/a	20.0	n/a
NIT-8072967	40	f	0.3	0.1	no	no	H/o surgery for massive disc herniation with cauda equina syndrome, post-traumatic stress disorder and anxiety disorder	yes	3.0	no	8.7	n/a
NIT-5579934	38	f	0.7	0.2	no	no	H/o infectious mononucleosis	no	1.5	no	12.0	n/a

Pseudonym	Age	Sex	Duration since 1. symp. (Y)	Duration since 1. diagnosis (Y)	Previous DMTs	Medication	Comorbidities	Relapse	EDSS	MRI active lesions	NOx serum (µM)	NOx CSF (µM)
NIT-8075595	29	f	0.3	0.1	no	n/a	Migraine	no	2.0	yes	28.2	n/a
NIT-8063169	29	m	0.1	0.0	no	no	H/o syphilis, vitamin D deficiency	yes	0.0	yes	26.7	n/a
NIT-7846668	36	f	0.0	0.0	no	no	Arachnoid cyst, epilepsy	no	n/a	n/a	7.2	n/a
NIT-8087889	33	f	0.0	0.0	no	no	n/a	yes	1.0	yes	41.1	n/a
NIT-4507026	57	m	0.6	0.0	no	Metoprolol, valsartan, metformin	Bilateral blindness due to ischemic diabetic maculopathy, h/o biliary pancreatitis and subsequent cholangitis due to choledocholithiasis associated with cholecystolithiasis, DM type II, PAD	yes	6.5	yes	23.2	10.6
NIT-8068254	35	m	1.1	0.0	no	no	no	no	0.0	n/a	8.6	4.1
NIT-8107689	35	f	0.1	0.0	no	no	S/p bilateral carpal tunnel syndrome	yes	2.0	no	2.3	2.9
NIT-7965198	25	f	6.1	0.0	no	Levothyroxine, folate	Migraine, hypothyroidism	no	2.0	n/a	3.4	n/a
NIT-3030561	57	f	9.7	0.0	no	no	Subclinical hypothyroidism, s/p hallux surgery, reflux disease, s/p ovarian cyst surgery	no	2.5	no	9.0	n/a
NIT-6217371	39	f	19.7	19.6	no	no	n/a	no	1.0	n/a	16.9	n/a
NIT-8092548	53	m	4.6	0.2	no	Gabapentin	Cushing's syndrome, obesity, h/o herpes zoster infection, h/o resection of pituitary adenoma	yes	2.0	no	3.1	n/a
NIT-6782991	53	m	0.9	0.0	no	Vitamin D, ramipril, hydrochlorothiazide, oxycodone, allopurinol, propionic acid, torasemide, pantoprazole	H/o radiculopathy, spondylarthrosis, h/o PTAs, suspected lymphedema, obesity, aHT, hyperuricemia, sleep apnea (CPAP therapy)	no	2.5	n/a	24.8	7.2
NIT-8062374	25	m	0.4	0.0	no	n/a	no	no	0.0	no	4.3	1.9
NIT-8102001	20	f	2.1	0.1	no	no	no	yes	1.0	yes	7.9	4.3
NIT-8088417	41	f	0.2	0.0	no	n/a	H/o right-sided iritis	yes	n/a	yes	5.0	4.4
NIT-8136921	22	f	0.3	0.2	no	no	no	no	1.0	yes	16.6	n/a
REB/K2-4917297	45	f	3.4	0.2	no	n/a	Hypothyroidism, hypercholesterolemia, migraine with aura, h/o bursitis, glaucoma	no	3.5	n/a	10.9	n/a
REB/K2-8134920	36	f	6.4	0.2	no	no	H/o gestational diabetes, chronic sinusitis	no	1.0	no	4.4	n/a
NIT-8181639	30	m	0.3	0.2	no	no	no	no	2.0	yes	4.7	n/a
NIT-3403428	30	m	0.5	0.2	no	n/a	Hypothyroidism	no	1.0	no	12.3	n/a
NIT-4348821	24	m	0.2	0.0	no	Vitamin B12	Vitamin D deficiency, Vitamin B12 deficiency	yes	2.0	yes	12.2	4.3
NIT-8087343	47	f	0.1	0.1	no	n/a	H/o vestibular neuritis, depression, hypothyroidism, cholecystectomy, h/o melanoma	yes	1.0	n/a	0.4	n/a
NIT-7172448	20	m	0.7	0.0	no	no	Recurrent nephrotic syndrome	yes	2.0	yes	7.6	6.0
NIT-7607967	20	m	0.1	0.0	no	no	no	yes	0.0	yes	4.0	n/a

Pseudonym	Age	Sex	Duration since 1. symp. (Y)	Duration since 1. diagnosis (Y)	Previous DMTs	Medication	Comorbidities	Relapse	EDSS	MRI active lesions	NOx serum (µM)	NOx CSF (µM)
NIT-6919392	54	f	1.3	0.0	no	Dapagliflozin , levothyroxine, valsartan, amlodipine, mirtazapine, liraglutid	Bipolar affective disorder, aHT, DM type II, hypothyroidism, dyslipidemia, suspected microadenoma of the pituitary gland, polyneuropathy, h/o malignant melanoma, h/o surgical treatment for a pilonidal abscess, h/o hysterectomy, bilateral finger polyarthrosis, muscular cervical spine syndrome, hallux valgus, chronic left ankle instability, bilateral metatarsalgia, bilateral high-frequency hearing loss	yes	n/a	no	10.5	2.7
NIT-8088474	31	f	3.3	3.3	no	n/a	VES, h/o two benign breast tumors	yes	3.0	n/a	n/a	6.1
NIT-8096613	34	f	5.1	5.1	no	contraception	Microprolactinoma, h/o eating disorders	no		no	6.0	n/a
NIT-7459605	28	m	0.8	0.3	no	n/a	H/o ossifying fibroma	no	1.0	n/a	6.2	n/a
NIT-8086905	22	f	0.4	0.3	no	no	Vitamin D deficiency and endometriosis	yes	2.0	no	2.0	n/a
NIT-8154425	37	f	0.7	0.6	no	Vitamin D	no	no	0.0	n/a	3.5	n/a
NIT-8163345	46	f	0.4	0.3	no	n/a	Hashimoto thyreoiditis	no	1.0	n/a	23.4	n/a
NIT-8134077	22	m	2.5	0.1	no	n/a	Vitamin D deficiency	no	1.0	yes	11.2	n/a
NIT-8166927	45	f	0.2	0.0	no	n/a	Vitamin D deficiency	yes	n/a	yes	12.9	5.1
NIT-8197809	50	f	0.3	0.0	no	no	H/o Lyme disease	yes	n/a	no	2.1	4.6
DUS-5332299	44	f	0.0	0.0	no	no	no	yes	1.0	yes	13.5	3.5
DUS-8189904	19	f	0.4	0.4	no	no	H/o miscarriage	no	1.0	n/a	2.8	n/a
DUS-8213607	29	f	0.8	0.7	no	no	Asthma, scoliosis	no	0.0	no	29.4	n/a
DUS-8197194	20	m	1.6	2.1	no	no	no	no	0.0	n/a	9.9	n/a
DUS-8176917	57	f	4.6	0.0	no	no	Hashimoto thyroiditis, migraine, aHT	no	2.5	n/a	16.6	n/a
DUS-8288859	30	f	0.1	0.0	no	no	no	yes	2.0	no	n/a	5.6
NIT-8048556	46	f	12.7	0.0	no	Valsartan	H/o herpes zoster, MCA aneurysm, aHT	no	n/a	no	n/a	3.0
NFL/R-7853181	41	m	0.1	0.0	no	no	no	yes	0.0	yes	n/a	2.4
NFL/R-7870851	24	f	0.1	0.0	no	no	Vitamin D deficiency	yes	1.0	yes	n/a	1.9
NFL/R-7863336	36	f	0.9	0.0	no	no	no	yes	0.0	yes	n/a	2.3
DUS-6586830	20	m	0.1	0.0	no	no	no	yes	1.0	yes	n/a	4.1
DUS-6224907	30	m	0.1	0.0	no	no	H/o traumatic brain injury	yes	n/a	yes	n/a	3.5

Supplementary Table S1 - Basic demographic and clinical data of RRMS patients

aHT - Arterial hypertension, CSF - Cerebrospinal fluid, COPD - Chronic obstructive pulmonary disease, DD - Differential diagnosis, DM - Diabetes mellitus, DMTs - Disease modifying therapies, EDSS - Expanded Disability Status Scale, H/o - History of, MCA - Middle cerebral artery, MRI - Magnetic resonance imaging, n/a - Not available, NOx - Nitrite/nitrate, PAD: peripheral arterial disease, PTA - Percutaneous transluminal angioplasty, RRMS - Relapsing remitting Multiple Sclerosis, Symp. - Symptoms, TEP - Total Endoprosthesis, VES - Ventricular extrasystoles, Y - Years.
