# Supplementary material for: The Use of Nitrosative Stress Molecules as Potential Diagnostic Biomarkers in Multiple Sclerosis
Source: Int J Mol Sci. 2024 Jan 8;25(2):787. doi: 10.3390/ijms25020787 (PMC10815836; doi:10.3390/ijms25020787)
Supplement: Supplementary file 1 [file ijms-25-00787-s001.zip › Suppl. Table S2.pdf]

Pseudonym	CSF leukocytes (/µl)	CSF albumin ratio	BCSFBD	IgG synthesis	IgG synthesis (%)	IgG ratio (x1/1000)	IgA synthesis	IgA synthesis (%)	IgA ratio	IgM synthesis	IgM synthesis (%)	IgM ratio	CSF OCBs	MRZ
NIT-7984596	n/a	n/a	n/a	n/a	n/a	n/a	n/a	n/a	n/a	n/a	n/a	n/a	yes	n/a
NIT-7936476	n/a	n/a	n/a	n/a	n/a	n/a	n/a	n/a	n/a	n/a	n/a	n/a	n/a	n/a
NIT-7968645	7.0	4.0	no	yes	25.0	3.5	no	0.0	1.1	yes	60.0	1.8	yes	neg.
NIT-7942539	13.0	6.5	yes	yes	27.0	6.5	no	0.0	2.4	no	0.0	1.1	yes	pos.
NIT-6047403	n/a	n/a	n/a	n/a	n/a	n/a	n/a	n/a	n/a	n/a	n/a	n/a	n/a	n/a
GEN-K-7888842	0.0	8.6	yes	no	0.0	4.2	no	0.0	2.9	no	0.0	0.5	no	neg.
NIT-7936341	10.0	7.2	yes	yes	43.0	9.4	yes	15.0	4.3	yes	36.0	2.7	yes	pos.
NIT-3276531	2.0	5.2	yes	yes	n/a	2.5	yes	65.0	6.7	no	0.0	0.6	yes	neg.
NIT-7990398	18.0	3.9	no	yes	84.4	16.8	no	0.0	1.6	yes	69.5	2.3	yes	pos.
NIT-7968168	13.0	n/a	n/a	n/a	n/a	n/a	n/a	n/a	n/a	n/a	n/a	n/a	yes	pos.
NIT-7993617	4.0	9.5	yes	yes	n/a	5.5	n/a	n/a	n/a	n/a	n/a	1.5	yes	neg.
NIT-7985475	5.0	6.3	no	yes	n/a	4.3	no	0.0	2.0	no	0.0	0.4	yes	neg.
REB/K1-6670439	n/a	n/a	n/a	n/a	n/a	n/a	n/a	n/a	n/a	n/a	n/a	n/a	yes	neg.
NIT-7999590	43.0	5.7	no	yes	69.9	13.4	no	0.0	1.6	no	0.0	0.6	yes	pos.
NIT-7588734	5.0	3.4	n/a	yes	n/a	2.7	n/a	n/a	1.0	n/a	n/a	0.7	yes	n/a
NIT-4311195	0.0	5.1	no	no	0.0	2.4	no	0.0	1.4	no	0.0	0.2	no	pos.
NIT-7689525	12.0	3.9	no	yes	43.6	4.6	no	0.0	0.9	yes	3.6	0.7	yes	n/a
NIT-6567357	3.0	5.3	no	yes	n/a	3.3	n/a	n/a	2.2	n/a	n/a	1.0	yes	pos.
NIT-7909971	n/a	n/a	n/a	n/a	n/a	n/a	n/a	n/a	n/a	n/a	n/a	n/a	n/a	n/a
NIT-5950698	n/a	n/a	n/a	n/a	n/a	n/a	n/a	n/a	n/a	n/a	n/a	n/a	yes	pos.
NIT-7958373	13.0	1.9	no	yes	71.0	4.2	yes	17.0	1.1	yes	87.0	2.7	yes	pos.
NIT-8058216	30.0	5.6	n/a	yes	63.6	10.9	yes	15.1	3.0	yes	54.2	2.5	yes	neg.
NIT-8041314	n/a	8.1	yes	yes	n/a	4.5	no	0.0	2.1	no	0.0	0.5	yes	neg.
NIT-8072967	5.0	n/a	n/a	yes	n/a	n/a	n/a	n/a	n/a	n/a	n/a	n/a	yes	pos.
NIT-5579934	5.0	3.8	n/a	yes	36.3	3.9	no	0.0	0.9	no	0.0	0.4	yes	n/a
NIT-8075595	n/a	n/a	n/a	n/a	n/a	n/a	n/a	n/a	n/a	n/a	n/a	n/a	yes	n/a
NIT-8063169	5.0	3.9	no	yes	n/a	2.2	no	0.0	0.9	no	0.0	0.3	yes	n/a
NIT-7846668	n/a	n/a	n/a	n/a	n/a	n/a	n/a	n/a	n/a	n/a	n/a	n/a	n/a	n/a
NIT-8087889	n/a	n/a	n/a	n/a	n/a	n/a	n/a	n/a	n/a	n/a	n/a	n/a	yes	neg.
NIT-4507026	9.0	11.3	yes	no	0.0	5.1	no	0.0	3.8	no	0.0	0.9	no	neg.
NIT-8068254	3.0	3.7	no	yes	n/a	1.8	no	0.0	0.9	no	0.0	0.4	yes	neg.
NIT-8107689	10.0	6.4	yes	yes	n/a	3.7	no	0.0	1.8	no	0.0	0.4	yes	neg.
NIT-7965198	7.0	1.6	no	yes	16.0	1.2	no	0.0	0.3	no	0.0	0.2	yes	pos.
NIT-3030561	8.0	4.2	no	yes	n/a	2.8	no	0.0	1.1	no	0.0	0.3	yes	n/a
NIT-6217371	n/a	n/a	n/a	n/a	n/a	n/a	n/a	n/a	n/a	n/a	n/a	n/a	yes	n/a
NIT-8092548	1.0	n/a	no	yes	n/a	n/a	n/a	n/a	n/a	n/a	n/a	n/a	yes	n/a

Pseudonym	CSF leukocytes (/µl)	CSF albumin ratio	BCSFBD	IgG synthesis	IgG synthesis (%)	IgG ratio (x1/1000)	IgA synthesis	IgA synthesis (%)	IgA ratio	IgM synthesis	IgM synthesis (%)	IgM ratio	CSF OCBs	MRZ
NIT-6782991	7.0	9.8	yes	yes	n/a	6.3	no	0.0	3.3	no	0.0	0.6	yes	neg.
NIT-8062374	8.0	4.3	yes	yes	n/a	2.2	no	0.0	1.3	yes	25.0	1.1	yes	n/a
NIT-8102001	5.0	4.7	no	yes	n/a	2.7	no	0.0	1.0	no	0.0	0.4	yes	neg.
NIT-8088417	3.0	6.4	no	no	0.0	2.9	no	0.0	1.5	no	0.0	0.2	no	neg.
NIT-8136921	n/a	n/a	n/a	n/a	n/a	n/a	n/a	n/a	n/a	n/a	n/a	n/a	yes	n/a
REB/K2-4917297	3.0	3.3	no	yes	n/a	2.7	n/a	n/a	n/a	n/a	n/a	n/a	yes	pos.
REB/K2-8134920	1.0	3.2	n/a	yes	n/a	2.6	n/a	n/a	n/a	n/a	n/a	n/a	yes	n/a
NIT-8181639	293.0	11.0	yes	yes	n/a	6.0	no	0.0	4.9	no	0.0	3.5	yes	n/a
NIT-3403428	4.0	5.0	no	no	0.0	2.9	no	0.0	1.4	no	0.0	0.5	no	neg.
NIT-4348821	13.0	7.0	yes	yes	n/a	4.7	no	0.0	2.2	no	0.0	0.5	yes	neg.
NIT-8087343	2.0	2.4	no	yes	n/a	1.5	no	0.0	0.5	no	0.0	0.1	yes	neg.
NIT-7172448	16.0	5.0	no	yes	13.0	4.0	no	0.0	1.8	yes	83.0	4.5	yes	neg.
NIT-7607967	17.0	4.2	no	yes	51.0	5.9	yes	16.0	2.2	no	0.0	0.8	yes	pos.
NIT-6919392	10.0	6.3	no	yes	13.0	5.3	no	0.0	1.6	no	0.0	0.4	yes	pos.
NIT-8088474	25.0	3.0	no	yes	61.0	4.9	yes	20.0	1.5	yes	35.0	0.8	yes	pos.
NIT-8096613	n/a	n/a	n/a	n/a	n/a	n/a	n/a	n/a	n/a	n/a	n/a	n/a	yes	n/a
NIT-7459605	0.0	n/a	n/a	n/a	n/a	n/a	n/a	n/a	n/a	n/a	n/a	n/a	yes	n/a
NIT-8086905	9.0	n/a	n/a	yes	62.2	n/a	yes	10.1	n/a	yes	45.3	n/a	yes	n/a
NIT-8154425	6.0	2.1	no	no	0.0	1.5	no	0.0	0.4	no	0.0	0.1	no	pos.
NIT-8163345	7.0	4.5	no	yes	47.1	5.9	no	0.0	1.3	no	0.0	0.2	yes	pos.
NIT-8134077	6.0	5.8	yes	yes	30.0	5.9	no	0.0	1.6	no	0.0	1.4	yes	neg.
NIT-8166927	9.0	4.8	no	yes	47.0	6.2	no	0.0	1.6	no	0.0	0.9	yes	n/a
NIT-8197809	3.0	2.5	no	yes	n/a	1.7	no	0.0	0.6	no	0.0	0.4	yes	neg.
DUS-5332299	0.0	4.6	no	no	0.0	2.3	no	0.0	1.3	no	0.0	0.2	no	n/a
DUS-8189904	14.0	2.6	no	yes	n/a	6.2	n/a	n/a	n/a	n/a	n/a	n/a	yes	n/a
DUS-8213607	5.0	5.2	no	yes	0.3	3.7	n/a	n/a	n/a	n/a	n/a	0.2	yes	pos.
DUS-8197194	70.0	7.8	yes	yes	n/a	4.9	n/a	n/a	2.7	n/a	n/a	0.6	yes	neg.
DUS-8176917	11.0	8.3	yes	yes	n/a	n/a	n/a	n/a	n/a	n/a	n/a	2.1	yes	pos.
DUS-8288859	10.0	7.8	yes	yes	n/a	5.2	no	0.0	2.6	no	0.0	0.6	yes	neg.
NIT-8048556	2.0	6.5	no	no	0.0	2.8	no	0.0	1.8	no	0.0	0.2	no	neg.
NFL/R-7853181	14.0	6.8	yes	yes	50.0	9.9	no	0.0	1.6	no	0.0	0.4	yes	pos.
NFL/R-7870851	16.0	10.3	yes	yes	n/a	8.8	no	0.0	3.9	no	0.0	1.2	yes	n/a
NFL/R-7863336	0.0	5.1	no	yes	n/a	3.8	no	0.0	1.5	no	0.0	0.6	yes	neg.
DUS-6586830	3.0	7.6	yes	yes	n/a	4.8	no	0.0	2.3	no	0.0	0.6	yes	n/a
DUS-6224907	6.0	8.1	yes	yes	n/a	5.0	yes	23.0	5.4	no	0.0	1.9	yes	n/a

Supplementary Table S2 - Basic CSF characteristics of RRMS patients

BCSFBD - Blood-CSF-barrier dysfunction, CSF - Cerebrospinal fluid, Ig - Immunoglobulin, MRZ - Antibody indices (AI) against measles, rubella, and varicella zoster virus. MRZ was defined 'positive' if at least two out of three AI were higher than 1.5, n/a - not available, OCBs - Oligoclonal bands, RRMS - Relapsing remitting Multiple Sclerosis.
