# Supplementary material for: The Use of Nitrosative Stress Molecules as Potential Diagnostic Biomarkers in Multiple Sclerosis
Source: Int J Mol Sci. 2024 Jan 8;25(2):787. doi: 10.3390/ijms25020787 (PMC10815836; doi:10.3390/ijms25020787)
Supplement: Supplementary file 1 [file ijms-25-00787-s001.zip › Suppl. Table S3.pdf]

Pseudonym	Age	Sex	Duration since 1. symp. (Y)	Duration since first diagnosis (Y)	Previous DMTs	Medication	Comorbidities	EDSS	MRI active lesions	NOx serum (µM)	NOx CSF (µM)
NIT-4536210	53	f	1.8	0.0	no	Vitamin D, vitamin B12	no	2.0	n/a	14.8	n/a
NIT-7984599	52	m	2.8	0.8	no	Magnesium	no	2.0	n/a	11.2	n/a
NIT-8018763	43	m	15.8	0.2	no	Baclofen	no	4.5	no	7.0	n/a
NIT-4823409	57	m	26.9	1.8	no	Fampridine, vitamin D, sertraline	H/o depressive episode	6.0	n/a	5.3	n/a
NIT-8058600	52	f	3.9	0.5	no	no	Sacroiliac joint arthritis/facet joint arthrosis	2.0	no	4.6	n/a
NIT-8054499	53	m	7.9	0.0	no	no	AHT, reflux esophagitis, spinal canal stenosis	1.0	no	6.6	n/a
NIT-8072688	50	f	5.9	0.0	no	Pregabalin	H/o sepsis, cholecysto-/choledocholithiasis with biliary pancreatitis, s/p cholecystectomy, s/p endoscopic mucosal resection of papillary adenoma, h/o left pelvic vein thrombosis, FAP, s/p laparoscopic proctocolectomy with ileo-anal pouch procedure	4.0	n/a	4.3	n/a
NIT-5107428	53	m	6.1	0.0	no	Rosuvastatin	Vitamin D deficiency, migraine with aura, h/o mitral valve reconstruction, paroxysmal atrial fibrillation, h/o polypectomy for suspicious inflammatory lesions	n/a	n/a	17.9	8.0
NIT-8077458	49	m	5.6	5.6	no	n/a	H/o alcohol use disorder and cannabis consumption	6.0	n/a	13.6	n/a
NIT-8145630	55	f	n/a	6.3	no	no	Amblyopia of left eye, h/o hysterectomy	2.0	no	5.9	n/a
NIT-8148987	44	m	1.2	0.2	no	no	n/a	4.5	n/a	5.9	n/a
NIT-8150661	54	f	27.4	0.3	no	no	n/a	3.0	n/a	7.4	n/a
NIT-5743011	43	f	4.8	0.1	no	no	Thumb saddle joint arthrosis	0.0	no	8.3	n/a
NIT-8194743	58	m	2.7	0.6	no	Vitamin B and D, magnesium	Small fiber neuropathy	1.0	no	11.3	n/a
NIT-5124471	52	f	1.0	0.5	no	n/a	H/o hysterectomy, Vitamin D deficiency	2.0	n/a	5.4	n/a
NIT-3077478	70	m	4.6	0.3	no	Aspirine, bisoprolol, ramipril, simvastatin	S/p myocardial infarction, CAD, aHT, macroangiopathy of the internal carotid arteries, degenerative spine changes, neuroforaminal stenoses, left kidney cysts, hypercholesterolemia, vitamin B12 deficiency, s/p melanoma, s/p cataract surgery	6.0	n/a	19.9	n/a

Supplementary Table S3 - Basic demographic and clinical data of PPMS patients

AHT - Arterial hypertension, CAD - Coronary artery disease, CSF - Cerebrospinal fluid, DMTs - Disease modifying therapies, EDSS - Expanded Disability Status Scale, FAP - Familial Adenomatous Polyposis, PPMS - Primary progressive Multiple Sclerosis, NOx - Nitrite/nitrate, MRI - Magnetic resonance imaging, Symp. - Symptoms, Y - Years.
