# Supplementary material for: The Use of Nitrosative Stress Molecules as Potential Diagnostic Biomarkers in Multiple Sclerosis
Source: Int J Mol Sci. 2024 Jan 8;25(2):787. doi: 10.3390/ijms25020787 (PMC10815836; doi:10.3390/ijms25020787)
Supplement: Supplementary file 1 [file ijms-25-00787-s001.zip › Suppl. Table S4.pdf]

Pseudonym	CSF leukocytes (/µl)	CSF albumin ratio	BCSFBD	IgG synthesis	IgG synthesis (%)	IgG ratio (x1/1000)	IgA synthesis	IgA synthesis (%)	IgA ratio	IgM synthesis	IgM synthesis (%)	IgM ratio	CSF OCBs	MRZ
NIT-4536210	n/a	n/a	n/a	yes	n/a	n/a	n/a	n/a	n/a	n/a	n/a	n/a	yes	pos.
NIT-7984599	33.0	7.9	yes	yes	n/a	n/a	n/a	n/a	n/a	n/a	n/a	n/a	yes	n/a
NIT-8018763	25.0	15.0	yes	yes	n/a	n/a	n/a	n/a	n/a	n/a	n/a	n/a	yes	pos.
NIT-4823409	n/a	n/a	n/a	n/a	n/a	n/a	n/a	n/a	n/a	n/a	n/a	n/a	n/a	n/a
NIT-8058600	5.0	5.1	no	yes	46.2	6,7	no	0.0	1.3	no	0.0	0.6	yes	n/a
NIT-8054499	1.0	6.3	n/a	yes	n/a	4.4	n/a	n/a	1.7	n/a	n/a	0.8	yes	n/a
NIT-8072688	4.0	4.3	n/a	yes	n/a	2.9	n/a	n/a	1.1	n/a	n/a	0.2	yes	pos.
NIT-5107428	22.0	11.0	yes	yes	56.0	20.3	no	0.0	3.2	no	0.0	2.3	yes	pos.
NIT-8077458	n/a	n/a	n/a	n/a	n/a	n/a	n/a	n/a	n/a	n/a	n/a	n/a	n/a	n/a
NIT-8145630	3.0	3.9	no	yes	21.7	3.3	no	0.0	1.1	no	0.0	0.2	yes	pos.
NIT-8148987	10.0	n/a	no	yes	n/a	11.0	n/a	n/a	1.7	yes	n/a	2.1	yes	n/a
NIT-8150661	9.0	12.0	yes	no	0.0	n/a	n/a	n/a	n/a	n/a	n/a	n/a	no	neg.
NIT-5743011	5.0	5.3	no	yes	n/a	3.6	n/a	n/a	1.8	n/a	n/a	0.6	yes	neg.
NIT-8194743	1.7	6.7	n/a	yes	n/a	4.7	n/a	n/a	2.0	n/a	n/a	1.0	yes	n/a
NIT-5124471	4.0	15.3	yes	yes	n/a	12.0	n/a	n/a	n/a	n/a	n/a	n/a	yes	neg.
NIT-3077478	25.0	8.8	n/a	yes	0.0	4.4	no	0.0	2.0	no	0.0	0.3	yes	neg.

Supplementary Table S4 - Basic CSF characteristics of PPMS patients

BCSFBD - Blood-CSF-barrier dysfunction, CSF - Cerebrospinal fluid, Ig - Immunoglobulin, MRZ - Antibody indices (AI) against measles, rubella, and varicella zoster virus. MRZ was defined 'positive' if at least two out of three AI were higher than 1.5, OCBs - Oligoclonal bands, PPMS - Primary progressive Multiple Sclerosis.
