# Supplementary material for: The Use of Nitrosative Stress Molecules as Potential Diagnostic Biomarkers in Multiple Sclerosis
Source: Int J Mol Sci. 2024 Jan 8;25(2):787. doi: 10.3390/ijms25020787 (PMC10815836; doi:10.3390/ijms25020787)
Supplement: Supplementary file 1 [file ijms-25-00787-s001.zip › Suppl. Table S5.pdf]

Pseudonym	Age	Sex	Previous DMTs	Medication	Comorbidities	MRI active lesions	NOx serum (µM)	NOx CSF (µM)
NIT-7990854	31	m	no	n/a	Scoliosis, thrombophlebitis	no	3.3	1.8
GEN-K-5434398	34	f	no	n/a	n/a	n/a	5.9	4.8
GEN-K-7897038	33	f	no	no	no	no	1.6	n/a
GEN-K-7250304	32	f	no	Propranolol, propylthiouracil	Graves' disease, platelet dysfunction, s/p splenic vein thrombosis, h/o gastric fundus varices treated with clipping, s/p resection of pancreatic pseudocyst, migraine	no	3.5	n/a
GEN-K-7895979	21	f	no	Levothyroxine	Hypothyroidism, obesity	no	0.5	2.9
GEN-K-7901346	28	f	no	n/a	no	n/a	8.7	n/a
GEN-K-3717612	54	f	no	Amlodipine, ramipril	S/p resection of a papillary urothelial carcinoma, neurodermatitis, hemochromatosis without clinical relevance, aHT	n/a	5.1	6.0
NIT-5501313	55	f	no	no	S/p resection of rectal carcinoma, ductal carcinoma in situ of the breast	n/a	8.1	n/a
NIT-7895802	29	f	no	Pregabalin, escitalopram	Depression, anxiety disorder	n/a	6.0	n/a
NIT-7799868	28	f	no	Hydroxychloroquine	Seronegative polyarthritis and tendonitis	n/a	8.0	4.3
NIT-6673248	33	f	no	Levothyroxine	Hereditary coagulation disorder, suspected mild factor XIII deficiency, suspected ACE gene polymorphism, h/o DVT, hypothyroidism, s/p left breast fibroadenoma	n/a	4.1	n/a
NIT-7818138	44	f	no	no	Suspected NAFLD, endometriosis, type C gastritis, vitamin D deficiency	n/a	1.9	n/a
NIT-3253011	31	f	no	Lamotrigine, perampanel	Epilepsy	n/a	6.0	n/a
NIT-8003463	21	f	no	no	Gilbert's syndrome	n/a	5.8	n/a
NIT-6174948	28	f	no	no	H/o varicose vein stripping, scoliosis, pectus excavatum	n/a	3.3	n/a
NIT-8082456	21	f	n/a	n/a	Right-sided peripheral facial palsy on the right, chronic hiccups	n/a	8.4	n/a
NIT-6468573	42	f	no	no	no	n/a	5.3	0.3
NIT-8085768	23	f	no	Salbutamol	Suspected C8 root syndrome DD left brachial plexus involvement, asthma	n/a	5.8	3.9
NIT-5430483	24	m	no	no	no	n/a	2.4	2.5
NIT-8088849	26	f	no	no	no	n/a	2.2	n/a
NIT-7328019	25	m	no	no	no	no	3.6	n/a
NIT-7305315	39	m	no	n/a	n/a	n/a	3.9	4.8
NIT-6339786	37	f	no	no	Latent hypothyroidism	n/a	7.0	n/a
NIT-8074584	51	f	no	Amlodipine, candesartan, levothyroxine, metoprolol, fluticasone furoate-umeclidinium-vilanterol, salbutamol	Depressive episode, h/o lumbar disc herniation, h/o pneumonia with pleural empyema, hypothyroidism, aHT	n/a	8.8	n/a
NIT-7068261	19	f	no	no	H/o right maxillary (zygomatic) and orbital floor fracture, h/o hyphema in the right eye following a contusion injury	n/a	1.7	2.2
NIT-4402605	23	f	no	Loratadine	n/a	n/a	5.5	n/a
NIT-6830004	52	f	no	Imipramine, enalapril, hydrochlorothiazide, pregabalin	AHT, depression, antelisthesis of L5 relative to S1, disc-ossification-related neuroforaminal and recessed stenosis, facet joint arthrosis	n/a	7.0	n/a
NIT-8085495	26	f	n/a	Levothyroxine	Hypothyroidism	n/a	6.0	n/a

Pseudonym	Age	Sex	Previous DMTs	Medication	Comorbidities	MRI active lesions	NOx serum (µM)	NOx CSF (µM)
DUS-4293525	20	f	no	no	no	no	n/a	3.6
DUS-8334942	36	f	no	no	no	no	n/a	3.1

Supplementary Table S5 - Basic demographic and clinical data of Soma patients

ACE - Angiotensin-converting enzyme, AHT - Arterial hypertension, CSF - Cerebrospinal fluid, DD - Differential diagnosis, DMTs - Disease modifying therapies, DVT- Deep vein thrombosis, EDSS - Expanded Disability Status Scale, H/o - History of, NAFLD - Non-alcoholic fatty liver disease, NOx - Nitrite/nitrate, MRI - Magnetic resonance imaging, Soma - Somatic symptom disorder, S/p - Status post.
