# Supplementary material for: The Use of Nitrosative Stress Molecules as Potential Diagnostic Biomarkers in Multiple Sclerosis
Source: Int J Mol Sci. 2024 Jan 8;25(2):787. doi: 10.3390/ijms25020787 (PMC10815836; doi:10.3390/ijms25020787)
Supplement: Supplementary file 1 [file ijms-25-00787-s001.zip › Suppl. Table S6.pdf]

Pseudonym	CSF leukocytes (/µl)	CSF albumin ratio	BCSFBD	IgG synthesis	IgG synthesis (%)	IgG ratio (x1/1000)	IgA synthesis	IgA synthesis (%)	IgA ratio	IgM synthesis	IgM synthesis (%)	IgM ratio	CSF OCBs	MRZ
NIT-7990854	<2	7.0	yes	no	0.0	3.4	no	0.0	2.7	no	0.0	0.7	no	neg.
GEN-K-5434398	4.0	3.4	no	no	0.0	1.5	no	0.0	0.6	no	0.0	0.2	no	neg.
GEN-K-7897038	<2	5.5	no	no	0.0	2.5	no	0.0	1.3	no	0.0	0.2	no	n/a
GEN-K-7250304	n/a	n/a	n/a	n/a	n/a	n/a	n/a	n/a	n/a	n/a	n/a	n/a	n/a	n/a
GEN-K-7895979	<2	3.5	no	no	0.0	1.7	no	0.0	0.8	no	0.0	0.1	no	n/a
GEN-K-7901346	<2.0	3.1	no	no	0.0	1.5	no	0.0	0.7	no	0.0	0.6	no	neg.
GEN-K-3717612	3.0	7.7	yes	no	0.0	4.3	no	0.0	2.8	no	0.0	0.4	no	neg.
NIT-5501313	<2.0	6.1	no	no	0.0	2.8	no	0.0	1.4	no	0.0	0.2	no	neg.
NIT-7895802	<2.0	3.3	no	no	0.0	1.4	no	0.0	0.7	no	0.0	0.2	no	neg.
NIT-7799868	2.0	2.9	no	no	0.0	1.3	no	0.0	0.6	no	0.0	0.2	no	neg.
NIT-6673248	<2.0	4.9	no	no	0.0	2.5	no	0.0	1.6	no	0.0	0.3	no	neg.
NIT-7818138	<2.0	3.1	no	no	0.0	1.6	no	0.0	0.8	no	0.0	0.3	no	neg.
NIT-3253011	2.0	3.6	no	no	0.0	1.5	no	0.0	1.0	no	0.0	0.2	no	neg.
NIT-8003463	3.0	n/a	no	n/a	n/a	n/a	n/a	n/a	n/a	n/a	n/a	n/a	no	neg.
NIT-6174948	<2.0	3.5	no	no	0.0	1.6	no	0.0	0.9	no	0.0	0.1	no	neg.
NIT-8082456	n/a	n/a	n/a	n/a	n/a	n/a	n/a	n/a	n/a	n/a	n/a	n/a	n/a	n/a
NIT-6468573	3.0	3.5	no	no	0.0	1.3	no	0.0	0.8	no	0.0	0.2	n/a	neg.
NIT-8085768	3.0	5.9	yes	no	0.0	3.2	no	0.0	1.8	no	0.0	0.9	no	neg.
NIT-5430483	<2.0	3.3	no	no	0.0	1.5	no	0.0	1.0	no	0.0	0.3	no	neg.
NIT-8088849	<2.0	3.1	no	no	0.0	1.4	no	0.0	0.7	no	0.0	0.1	no	n/a
NIT-7328019	4.0	5.8	yes	no	0.0	2.4	no	0.0	1.5	no	0.0	0.2	no	n/a
NIT-7305315	<2.0	4.2	no	no	0.0	2.1	no	0.0	1.1	no	0.0	0.2	no	n/a
NIT-6339786	<2.0	3.7	no	no	0.0	1.6	no	0.0	1.0	no	0.0	0.3	no	neg.
NIT-8074584	<2.0	6.5	no	no	0.0	2.8	no	0.0	1.4	no	0.0	0.2	no	n/a
NIT-7068261	<2.0	2.4	no	no	0.0	1.1	no	0.0	0.5	no	0.0	0.2	no	neg.
NIT-4402605	<2.0	4.6	no	no	0.0	2.1	no	0.0	1.2	no	0.0	0.1	no	n/a
NIT-6830004	<2.0	3.7	no	no	0.0	1.4	no	0.0	0.9	no	0.0	0.1	no	neg.
NIT-8085495	n/a	n/a	n/a	n/a	n/a	n/a	n/a	n/a	n/a	n/a	n/a	n/a	n/a	n/a
DUS-4293525	3.0	4.2	no	n/a	n/a	1.9	n/a	n/a	1.1	n/a	n/a	0.2	no	neg.
DUS-8334942	<2.0	2.5	no	no	0.0	1.3	no	0.0	0.5	no	0.0	0.1	no	neg.

Supplementary Table S6 - Basic CSF characteristics of Soma patients

BCSFBD - Blood-CSF-barrier dysfunction, CSF - Cerebrospinal fluid, Ig - Immunoglobulin, MRZ - Antibody indices (AI) against measles, rubella, and varicella zoster virus. MRZ was defined 'positive' if at least two out of three AI were higher than 1.5, OCBs - Oligoclonal bands, Soma - Somatic symptom disorder.
